# The complete chloroplast genome of *Epimedium elachyphyllum* Stearn (Berberidaceae), an endangered species endemic to China

**DOI:** 10.1080/23802359.2020.1721357

**Published:** 2020-02-03

**Authors:** Fengmei Suo, Xiang Liu, Cheng Zhang, Chaoqun Xu, Guoan Sheng, Baolin Guo

**Affiliations:** a Institute of Medicinal Plant Development, Chinese Academy of Medical Science, Peking Union Medical College, Beijing, People’s Republic of China;; b Chongqing Academy of Chinese Materia Medica, Chongqing, People’s Republic of China

**Keywords:** Chloroplast genome, *Epimedium elachyphyllum* Stearn, Berberidaceae

## Abstract

*Epimedium elachyphyllum*, which belongs to Berberidaceae, is only distributed in Guizhou province of China. In this study, the complete chloroplast (cp)genome of *E*. *elachyphyllum* was sequenced and assembled. The circular genome is 157,201 bp in length, which comprises of a large single-copy region (LSC, 88,519 bp), a small single-copy region (SSC, 17,042 bp), and a pair of inverted repeat regions (IRa and IRb, 25,820 bp). The genome of *E*. *elachyphyllum* contains 112 unique genes, of which 78 protein-coding genes, 30 tRNA genes, and four rRNA genes. Phylogenetic analysis based on 18 complete cp genome sequences indicated that *E*. *elachyphyllum* was closely related to E. dolichostemon.

*Epimedium elachyphyllum*, an endangered species endemic to China, is only distributed in a small area in the Guizhou province of China. Due to habitat loss, *E*. *elachyphyllum* has been classified as endangered in “China Species Red List” (Ministry of Environment Protection of the People’s Republic of China 2013). *Epimedium* L. family contains about 60 species intermittently distributed in the Eurasia between Europe and eastern Asia. (Xu et al. 2019). The major bioactive components are flavonoid glycosides in Epimedii Folium (Wang et al. 2007; Ma et al. 2011). The therapeutic effects of *E*. *elachyphyllum* was the same as *E*. *sagittatum* (sieb. Et zucc.) Maxim (He and Zhang 1994). Previous studies showed that the classification and phylogeny were not completely clear in the *Epimedium* family (Guo et al. 2018). It was reported that the chloroplast genome has a highly conserved sequence ranging approximately 150k bp, which proves more variation information to discriminate closely related plants (Li et al. 2015). Up to now, the complete chloroplast (cp) genome of five *Epimedium* species have been reported (Zhang et al. 2016; Liu et al. 2019). So far, no data are available regarding the chloroplast genome of *E*. *elachyphyllum*. In this study, we assembled and characterized the complete chloroplast (cp) genome of *E*. *elachyphyllum* for the first time. The complete chloroplast (cp) genome sequence of *E*. *elachyphyllum* is a valuable resource for further studies, including species identification and genetic evolution in the family Berberidaceae.

In this study, *E*. *elachyphyllum* sample was collected from the Songtao County of Guizhou province in China (28^°^26′N, 108°42′E). A voucher specimen (Zhang218) was deposited at the Herbarium of the Institute of Medicinal Plant (IMPLAD), Beijing, China. Total genomic DNA was extracted from the fresh leaves of *E*. *elachyphyllum* using the modified CTAB method (Doyle and Doyle 1987). The high-quality DNA was sheared to the size of 300 bp for the shotgun library construction. The sequencing was performed on an Illumina Novaseq PE150 platform (Illumina Inc, San Diego, CA), and 150 bp paired-end reads were generated. The filtered reads were assembled into the complete chloroplast (cp) genome using the program GetOrganelle v1.5 (Jin et al. 2018) with *E*. *acuminatum* chloroplast genome (GenBank accession number: NC_029941) as a reference. The annotation of chloroplast genome was conducted through the online program CPGAVAS 2 (Shi et al. 2019), followed by manual correction if required. The annotated genomic sequence has been registered in GenBank with an accession number (MN873562).

The complete chloroplast (cp) genome of *E*. *elachyphyllum* is 157,201 bp in length and shows a typical quadripartite structure, which consists of a large single-copy region (LSC, 88,519 bp), a small single-copy region (SSC, 17,042 bp), and a pair of inverted repeat regions (IRa and IRb, 25,820 bp). The genome of *E*. *elachyphyllum* contains 112 unique genes, of which 78 protein-coding genes, 30 tRNA genes, and 4 rRNA genes. The total GC content of *E*. *elachyphyllum* chloroplast genome is 38.77%, while the corresponding GC content of LSC, SSC, IR regions are 37.36%, 32.78%, and 43.16%, respectively. The intron-exon structure analysis indicated that eleven protein-coding genes and seven tRNA genes contained one intron, while four genes (ycf3, clpP and rps12) had two introns.

To identify the phylogenetic relationship of *E*. *elachyphyllum*, 15 complete chloroplast (cp) genomes of Berberidaceae species were used to reconstruct a maximum-likelihood (ML) phylogenetic tree using RAxML v8.2.10 (Stamatakis 2014), with *Aconitum angustius* and *Aconitum austrokoreenseas* as the outgroup. Phylogenetic analysis indicated that *E*. *elachyphyllum* is closely related to *E*. *dolichostemon* (Figure 1). The published *E*. *elachyphyllum* chloroplast genome will provide useful information for phylogenetic and evolutionary studies in Berberidaceae.

**Figure 1.**
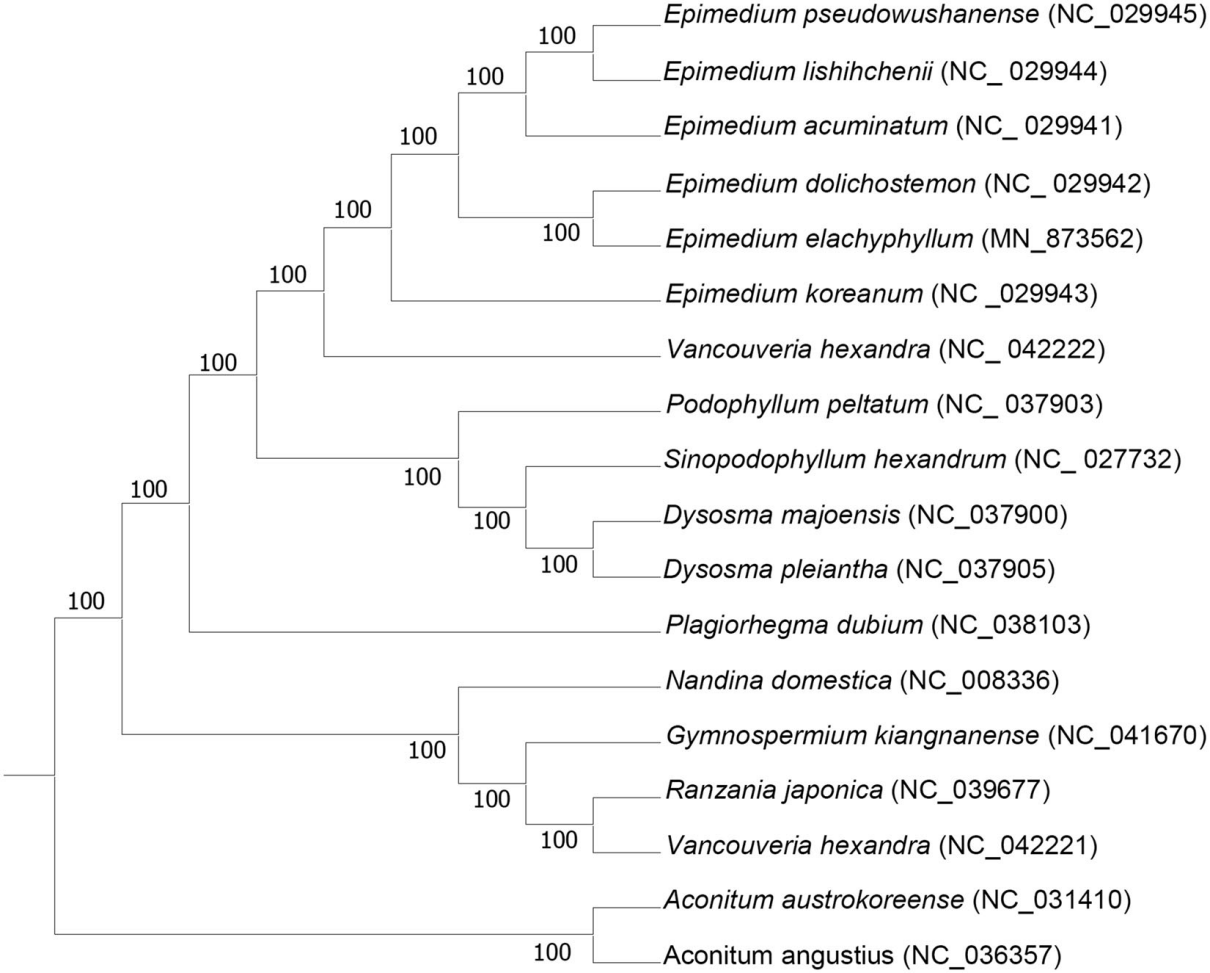
ML phylogenetic tree inferred from 18 complete cp genomes.
